# Methodology for Hydrogen-Assisted Fatigue Testing Using In Situ Cathodic Charging

**DOI:** 10.3390/ma18020339

**Published:** 2025-01-14

**Authors:** Kai Donnerbauer, Timo Nickel, Matthias von Pavel, Johannes L. Otto, Lars Gerdes, Julian Rozo Vasquez, Lars A. Lingnau, Alexander Koch, Frank Walther

**Affiliations:** 1 Chair of Materials Test Engineering (WPT), TU Dortmund University, Baroper Str. 303, D-44227 Dortmund, Germanylars.lingnau@tu-dortmund.de (L.A.L.);; 2 Institute for Research and Transfer e.V. (RIF), Joseph-von-Fraunhofer-Str. 20, D-44227 Dortmund, Germany

**Keywords:** fatigue engineering, AISI 4140, in situ cathodic hydrogen charging, electrochemical hydrogen charging, hydrogen embrittlement, fractography

## Abstract

With hydrogen being a promising candidate for many future and current energy applications, there is a need for material-testing solutions, which can represent hydrogen charging under superimposed mechanical loading. Usage of high purity gaseous hydrogen under high pressure in commercial solutions entails huge costs and also potential safety concerns. Therefore, a setup was developed utilizing a customized electrochemical charging cell built into a dynamic testing system. With this setup, two heat treatment states of AISI 4140 (DIN 1.7225, 42CrMo4) with varying yield and ultimate tensile strength were characterized in constant amplitude tests. S-N (Woehler) curves differ between heat-treated states, and when comparing testing in air with in situ cathodic hydrogen-charged specimens, hydrogen proves to be detrimental to the material properties. For both states considered, the presence of hydrogen leads to a reduction in fatigue life. Fractographic analyses by scanning electron microscopy reveals that for in situ cathodic hydrogen-charged specimens, the crack initiation mechanisms change for the higher strength heat treatment state.

## 1. Introduction

With the increasing role of hydrogen in various industries, particularly in the context of clean energy and environmental sustainability [1], there is an emerging and significant need for testing structural materials under the presence of hydrogen [2]. With this trend towards a hydrogen economy, the infrastructure for production, storage and distribution will expand significantly. Hence follows, that many structural materials will be used in applications where mechanical loading and hydrogen charging are superimposed [3]. However, hydrogen is detrimental to the mechanical properties of many metallic materials [4]. Steels, in general, but especially medium- and high-strength quenched and tempered martensitic steels are endangered [5,6]. An uptake of hydrogen is known to reduce ductility up to the possibility of brittle fractures even at concentrations of around only 1 mass ppm [7,8]. Also, under tensile stress the hydrogen desorption is increased [9]. Especially, the uptake of dislocation-trapped hydrogen is increased according to diffusion-coupled finite element analysis [10]. Hydrogen also leads to reduced fatigue performance [11,12,13,14,15]. In general, uniaxial fatigue data are recorded by constant amplitude testing in servohydraulic, electrodynamic, or resonance testing systems, whereby resonance testing systems are used in the very high cycle-fatigue regime since they can reach much higher testing frequencies. Though many test methods have been developed to measure decreasing mechanical properties under hydrogen [16], currently available standards (ASTM G129, ASTM F1624 and ISO 16573 [17,18,19]) merely cover incremental step test [20], constant load test and slow strain rate test [21]. In all tests, the mechanical load is applied as quasi-static or static, but not cyclic, which is often present under service in relevant applications. Fracture surfaces on tempered martensitic steel caused by hydrogen embrittlement can exhibit transgranular quasi-cleavage (QC) features but also intergranular (IG) features [22,23,24,25]. The underlying mechanisms are still discussed and can occur simultaneously [22,26,27,28], but brittle mechanisms of IG fractures such as the lowering of cohesive energies [29] and ductile mechanisms of QC fractures like hydrogen-enhanced localized plasticity [30] have been proposed. QC fractures can show river-like patterns on the facets of transgranular fracture portions [31]. IG fractures occur along prior austenite grain boundaries (PAGB) and are commonly accompanied by secondary cracks [32,33]. The influence of PAGB has been researched extensively. For martensitic steels, it has been reported that more hydrogen will be accumulated around PAGB with increasing initial dislocation density [34]. Mechanical testing can be performed using specimens pre-charged electrochemically in a liquid electrolyte or under a high-purity high-pressure gaseous hydrogen atmosphere [35]. Pre-charged specimens do not fully represent service conditions, since mechanical loading and hydrogen charging happen simultaneously during service. There are already existing testing setups to apply mechanical loading during hydrogen charging under gaseous hydrogen atmospheres, but these are very cost-intensive, hardly available and developed for specific-use cases, which is due to the properties and dangers of pressurized hydrogen [36,37]. If specimens are pre-charged, hydrogen concentration in lattice decreases greatly with increasing testing frequency. This effect is much less pronounced in an open system, where hydrogen is provided constantly [38]. Therefore, in situ testing methodologies enabling broad frequency ranges and testing conditions similar to servohydraulic testing systems would be desirable. With an increase in the hydrogen economy as it is currently occurring, a need for efficient testing setups for the determination of material properties during simultaneous hydrogen charging and mechanical loading arises. Electrochemical hydrogen charging is commonly applied followed by mechanical testing, but due to its relatively easy experimental setup, it has also been applied during quasi-static mechanical testing [39,40]. In this work, an efficient setup is presented allowing electrochemical hydrogen charging inside a universal fatigue testing system under dynamic loading conditions realizing testing frequencies of 10 Hz. However, the limitation in terms of testing frequencies depends only on the servohydraulic testing system and is not decreased due to the charging cell. Due to the very low hydrogen volume in comparison to testing under high-pressure gaseous hydrogen, no additional safety equipment was necessary. The components are also widely available without high costs. Therefore, the method can significantly improve the accessibility of dynamic mechanical testing under the influence of hydrogen.

## 2. Materials and Methods

### 2.1. Material and Heat Treatment

Steel AISI 4140 (DIN 1.7225, 42CrMo4), manufactured from RIVA Stahl GmbH, Henningsdorf, Germany was chosen as a model material. Chemical composition determined by optical emission spectroscopy of the material was in accordance with DIN EN ISO 683-2 and is given in Table 1 [41].

**Figure 1:**
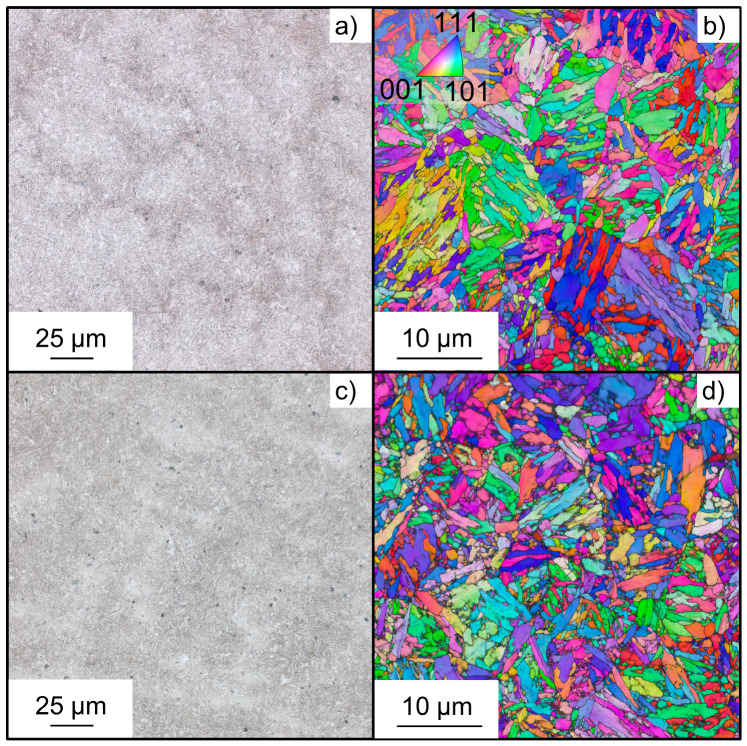
Optical microscopy images and orientation mappings by electron backscatter diffraction of (**a**,**b**) medium strength (MS) and (**c**,**d**) high strength (HS) heat treatment states.

To investigate the influence of strength on hydrogen susceptibility under fatigue loading, two different material states obtained by means of heat treatment were studied. The austenitizing temperature was 840 °C under a nitrogen atmosphere and kept for 30 min, followed by quenching in water for both conditions. Two different tempering temperatures of 650 °C and 400 °C were used for 30 min in air. These temperatures are subsequently referred to as medium strength (MS) and high strength (HS). Optical microscopy and analysis by electron backscatter diffraction (EBSD) in horizontal cross-sections are provided in Figure 1. Specimens for EBSD underwent metallographic preparation including cutting, embedding, grinding, and polishing up to a 0.05 µm colloidal silica suspension. For optical microscopy, alcoholic nitric acid was used as an etchant. EBSD images were acquired in a field emission gun scanning electron microscope Tescan Mira III (Tescan, Brno, Brno, Czech Republic) equipped with APEX EDAX Velocity Pro detector (AMETEK Inc., Berwyn, PA, USA) at 25 kV acceleration voltage and a step size of 50 nm.

Both material states exhibit a tempered martensitic microstructure with fine needle-like grains and prior austenite grains being recognizable by orientation contrast. The quasi-static properties in air were determined by tensile testing and are summarized as a reference for further fatigue testing in Table 2. Also, the Vickers hardness is given.

After heat treatment, material states differed greatly in quasi-static strength and hardness with values of HS being approximately 60% higher than for MS.

### 2.2. Fatigue Specimens and Preparation

**Figure 2:**
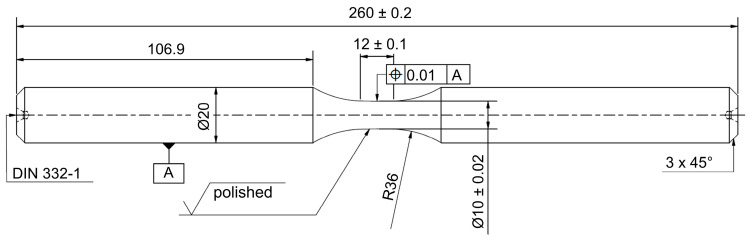
Unnotched fatigue testing specimen geometry.

Fatigue tests were carried out using unnotched specimens with a diameter of 10 mm and a length of 12 mm in the gauge section. The specimen geometry is shown in Figure 2. After turning, specimens were polished mechanically. To define a constant area for electrochemical charging, specimens were then coated with the polyurethane varnish Urethan 71 by CRC Industries (CRC Industries Inc., Horsham, PA, USA). However, the gauge length was masked during coating in order to ensure a metallic surface in the smallest cross-section for hydrogen entry.

### 2.3. Experimental Procedure

**Figure 3:**
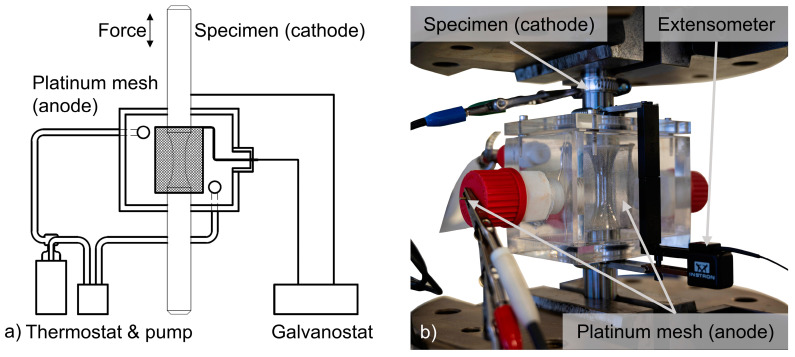
In situ cathodic hydrogen charging inside a fatigue testing system. (**a**) Schematic sketch, (**b**) real experimental setup.

All fatigue tests were performed with the servohydraulic fatigue testing system Instron 8802 (Instron, Darmstadt, Germany) with a maximum load capacity of 250 kN. R = 0.1 was chosen as the stress ratio, to ensure that the material is constantly under tensile stress because of the already-investigated stress dependence of hydrogen diffusion. The testing frequency f was constant at 10 Hz and runouts were defined at 3 × 10^6^ cycles, because fracture occurred beyond the common limit of 2 × 10^6^ cycles. A two-electrode electrochemical system in a custom build polymethyl methacrylate (PMMA) cell connected to galvanostat Gamry 1000 A (Gamry Instruments, Warminster, PA, USA) was mounted around the fatigue specimen, as shown in Figure 3.

**Figure 4:**
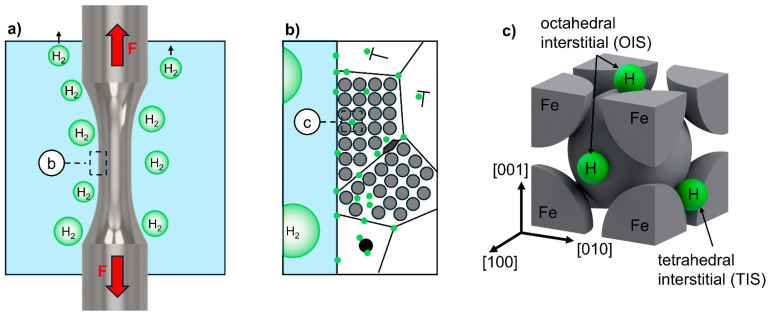
Scale-bridging schematic representation of in situ electrochemical hydrogen charging: (**a**) Macroscopic scale with specimen under cyclic tension stress and molecular hydrogen in form of gas bubbles; (**b**) detail of polycrystalline specimen surface with adsorbed atomic hydrogen and diffusion into the metallic lattice with trapped hydrogen at typical defects; and (**c**) 3D representation of an iron bcc unit cell with hydrogen in interstitial tetrahedral and octahedral sites.

The electrolyte, which is a 3.5 wt.% NaCl solution, was prepared using deionized water and analytical grade NaCl. Usage of solutions of NaCl are in accordance with ISO 16573 and have been applied by various authors before [42,43,44,45]. Due to the cathodic corrosion protection, no corrosive effects such as pitting were observed macroscopically or fractographically in this work. The electrolyte was circulated and tempered to 20 °C by a thermostat. The susceptibility to hydrogen-assisted fatigue was evaluated by in situ cathodic hydrogen charging with a constant current density of 0.075 A/mm^2^ with a platinum mesh electrode surrounding the gauge length as the counter and the fatigue specimen as the working electrode. The charging time is dependent on the time until the specimen fracture, since hydrogen charging was only applied during mechanical loading. The charging process during the fatigue loading is schematically depicted in more detail across length scales in Figure 4.

Most of the hydrogen provided by the electrochemical charging recombines into molecular hydrogen and escapes in the form of gas bubbles, Figure 4a. However, some of the hydrogen is adsorbed on the specimen surface as atomic hydrogen, which can diffuse into the polycrystalline metal lattice. However, it can be trapped at metallic defects such as pores, grain boundaries, triple grain boundaries, dislocations, stacking faults and precipitates, as shown in Figure 4b. Integration into the body-centered cubic (bcc) metal lattice as mobile hydrogen can occur interstitially in tetrahedral (TIS) or octahedral (OIS) form, as shown in Figure 4c.

## 3. Results and Discussion

### 3.1. Constant Amplitude Testing

**Figure 5:**
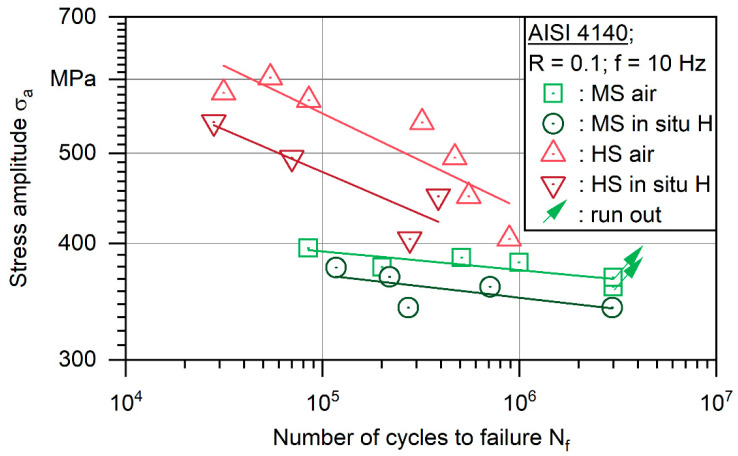
S-N data of heat-treated steel AISI 4140 (DIN 1.7225, 42CrMo4) in medium strength (MS) and high strength (HS) states, tested in air and in situ cathodic hydrogen charging conditions.

Figure 5 illustrates the results of constant amplitude tests in S-N curves representing stress amplitude σ_a_ over the number of cycles to failure N_f_. For both heat treatment states, testing in air leads to higher N_f_ than testing with in situ cathodic hydrogen charging. On average, comparing power law fits, the S-N curve of HS state in air is 15% higher compared to the in situ cathodic hydrogen charging in the region where experimental data were recorded. On the other side, the fit for the MS state in air is only 7.2% higher, meaning that the hydrogen-assisted fatigue was more severe for the higher strength material. However, it has to be stated that fit curves for the HS state show a similar slope, thus, being independent on the number of cycles to failure. In the MS state, the distance between curves increases slightly with a higher number of cycles to failure, which indicates an increasing effect of hydrogen in higher cycle fatigue regimes. This can be interpreted as a higher hydrogen concentration being needed to negatively influence the lower strength heat treatment. This is in accordance with the known correlation between strength and susceptibility to hydrogen. Also, there is no decline of the S-N curve with an increasing number of cycles to failure, as would be expected, if anodic corrosion processes decreased the fatigue life. The constant slope supports the fact that material degradation, due to presence of hydrogen, is the reason for the reduction in fatigue life.

For the HS state, the S-N curve is much steeper in general. However, even at σ_max_ = 0.62·R_e_ no runouts were achieved, whereas at the MS state, a runout occurred at maximum stress σ_max_ = 0.935·R_e_. All crack initiations in air for both states and in situ cathodic hydrogen charging for MS were detected to occur at inclusions. For the HS state, crack initiation happened at specimen surfaces and at inclusions close to the surface under hydrogen charging. Due to the nature of cathodic charging, this corresponds to the highest local hydrogen concentrations and also underlines the increasing susceptibility with increasing strength. Since most cracks were initiated at inclusions, the scatter of data can be explained by the varying size of those. Interestingly, fracture occurred at 2.96 × 10^6^ at σ_a_ = 340 MPa for the MS state under cathodic hydrogen charging beyond the commonly used runout limit of 2 × 10^6^. In contrast, two runouts were recorded at higher stress levels for testing in air. This implies that even in a very high cycle-fatigue regime, fracture will occur, if hydrogen is offered.

### 3.2. Fractographic Investigations

**Figure 6:**
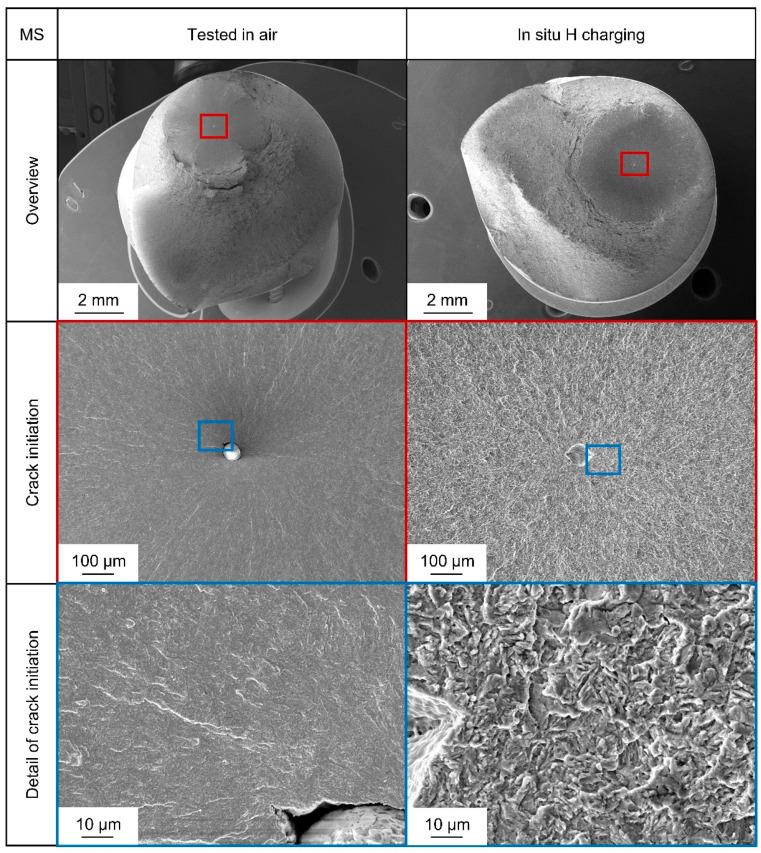
Comparison of fracture surfaces of two representative specimens of heat-treated steel AISI 4140 (DIN 1.7225, 42CrMo4) in medium strength (MS) state, tested in air (on the left) at σ_a_ = 380 MPa and in situ cathodic hydrogen charging condition (on the right) at σ_a_ = 360 MPa.

In order to obtain information about the failure mechanisms involved, the specimens were fractographically examined in the scanning electron microscope Tescan Mira III (Tescan, Brno, Brno, Czech Republic). Figure 6 shows typical fracture surfaces for the medium strength heat treatment. Fracture initiation always occurred at inclusions, which can be identified as aluminum oxide or magnesium oxide and can be found either as shown inside the specimen bulk or close to the surface. When comparing the fracture surfaces for MS, no systematic differences between testing in air and testing under in situ cathodic hydrogen charging regarding crack initiation at inclusions could be observed. Nevertheless, the morphology of fracture surfaces around the inclusions changed. Both show transgranular QC morphologies, but under hydrogen charging, the surface is more faceted and shows river-like patterns. The most likely mechanism for the change in morphology of the transgranular fracture surfaces is hydrogen-enhanced localized plasticity. If crack initiation takes place at an inclusion in bulk, a corrosive influence can be ruled out. Therefore, material degradation due to hydrogen diffusion towards the stress concentration at the inclusion is the explanation for the observed reduction in fatigue lifetime, even for steel with an ultimate tensile strength below 1000 MPa.

**Figure 7:**
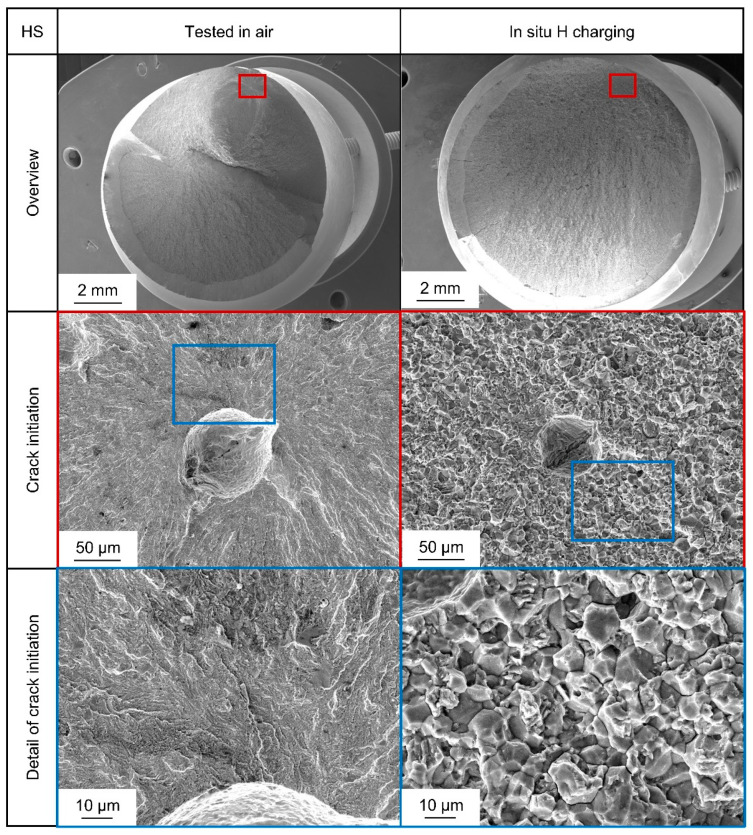
Comparison of fracture surfaces of two representative specimens of heat-treated steel AISI 4140 (DIN 1.7225, 42CrMo4) in a high strength (HS) state, tested in air (on the left), at σ_a_ = 450 MPa, and in situ cathodic hydrogen charging condition (on the right) at σ_a_ = 450 MPa.

Figure 7 shows typical fracture surfaces for the HS state, where significant differences to medium strength and also between testing in air and testing under in situ cathodic hydrogen charging are observable. Fracture initiation in air again always occurred at inclusions. The fatigue fracture surfaces show a comparable fracture surface to Figure 6 for testing in air with transgranular fracture initiated at an inclusion. When hydrogen is provided, electrochemically fractured surfaces change and show IG fracture portions along PAGB, together with QC transgranular regions. Since for the HS state an increased uptake of hydrogen around PAGB can be expected because of higher initial dislocation density [34], brittle IG fracture portions only for this state are a coherent result. Crack initiation for the specimen in Figure 7 occurred in bulk at an inclusion even though it occurred close to the surface. Since a crack initiation due to corrosion would have to start at the surface, it can be concluded that the local stress concentration of the inclusion, together with the presence of hydrogen, led to hydrogen-assisted failure.

**Figure 8:**
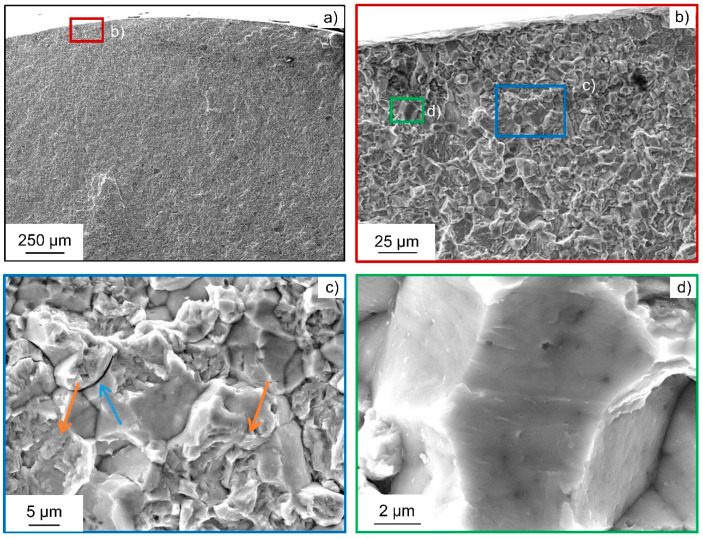
Representative fracture surface of the heat-treated steel AISI 4140 (DIN 1.7225, 42CrMo4) in high-strength state subjected to in situ cathodic hydrogen charging at σ_a_ = 540 MPa: (**a**) Overview of the crack initiation area close to the specimen surface; (**b**) crack initiation area showing intergranular and transgranular portions; (**c**) evidence of hydrogen-induced cracks by facets (orange arrows) and secondary cracks along grain boundaries (blue arrows); and (**d**) detail of an intergranular crack.

These transgranular QC fracture portions also change in morphology, as it is typical for hydrogen-induced fracture due to hydrogen-enhanced localized plasticity [23,24]. This is very visible at higher magnifications. Moreover, secondary cracks along grain boundaries become visible, which is, together with brittle intergranular fracture surfaces, another characteristic feature for hydrogen-induced fracture by a hydrogen-enhanced decohesion mechanism, as shown in Figure 8. Therefore, both mechanisms seem to be active at the same time for the HS state. Synergistic activity of both mechanisms has been reported before [46].

**Figure 9:**
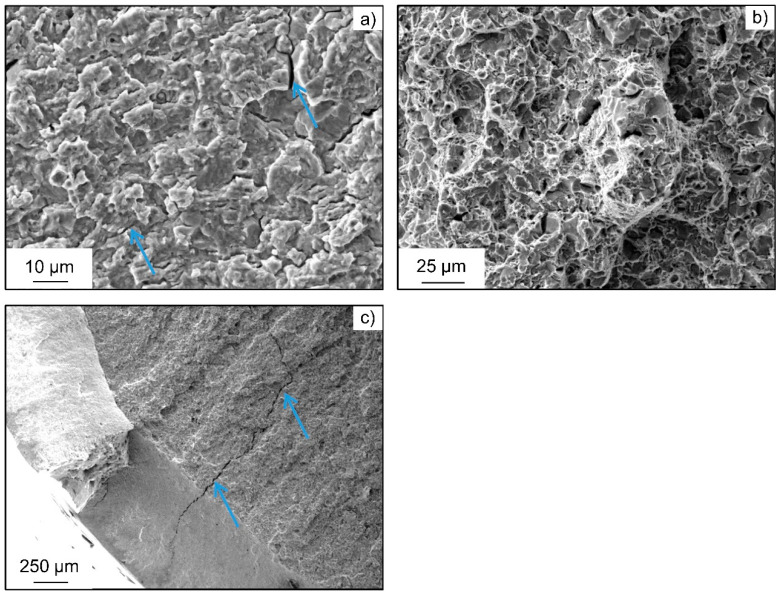
Representative fracture surface of the heat-treated steel AISI 4140 (DIN 1.7225, 42CrMo4) in high-strength state subjected to in situ cathodic hydrogen charging at σ_a_ = 540 MPa: (**a**) transient area with secondary cracks (blue arrows) and quasi cleavage fracture surface; (**b**) partly ductile dimple fracture in the center of the specimen; and (**c**) secondary cracks on the opposite side of the crack initiation area near the specimen surface (blue arrows).

In summary, regardless of the location of crack initiation (surface or inclusion), for the HS treatment, the fracture initiation mechanism changes from transgranular to a mixture of transgranular QC, with high IG portions. As with the MS specimens, the transgranular portions change to a more faceted morphology. Figure 9 shows insights about the remaining fracture. There is a transient area, where intergranular features are not present anymore, but secondary cracks remain, and facets become less present. Figure 9b shows a partly ductile dimple fracture surface in the specimen center. Since hydrogen concentration is expected to be the lowest in the specimen center, it is consistent that the most ductile fracture portion could be detected there. Quite prominent secondary cracks were found again towards the specimen surface.

## 4. Discussion, Conclusions and Outlook

This work presents a methodology for hydrogen-assisted fatigue testing using in situ cathodic charging. Results agree with the expected behavior, showing a more severe degradation for the high-strength heat treatment compared to the lower strength one, when specimens are charged with hydrogen under simultaneous fatigue loading. The fractographic analysis revealed a significant change in fracture morphology. Only for specimens with high-strength heat treatment, intergranular fractures are dominant, due to the hydrogen-enhanced decohesion mechanism, while no intergranular fracture occurred for specimens tested in air. Crack initiation happened mostly at inclusions, ruling out an influence of corrosion, but a reduction in fatigue lifetime was still observed even at a testing frequency of 10 Hz for a material with an ultimate tensile strength below 1000 MPa. This means that even comparatively low strength steel grades should be investigated in detail, if they are exposed to a possible hydrogen uptake. If the results are to be transferred to another grade, a comparable chemical composition should be present, but most importantly, yield and ultimate strength should not differ, since a clear influence was shown.

The developed setup is highly versatile, allowing easy implementation into other testing systems without the necessity to fulfill additional conditions, aside from those inherent to the fatigue testing system itself. This flexibility underscores the potential for broad application across various materials and testing conditions. In comparison to testing with gaseous hydrogen under high pressure, the setup is easier to handle, safer, cheaper, and therefore more accessible.

Future work should expand the current S-N and fractographic data, allowing a more statistically robust quantitative assessment. More fractographic data could allow quantitative separation of intergranular and transgranular features by image segmentation. The method should be applied to a wider array of electrolytes and materials to further validate the methodology. Steels with a face-centered cubic crystal structure like AISI 316, which is used in hydrogen storage and transport applications, would be interesting candidates. Implementation into a resonance fatigue testing system would allow insights into very high cycle-fatigue regime under hydrogen loading. The influence of low loading levels at a very high number of cycles to failure and, therefore, comparatively long in situ charging times, could be studied in resonance testing systems. Additionally, a comprehensive comparison of data from tests with gaseous hydrogen would provide valuable insights and a deeper understanding of hydrogen embrittlement mechanisms across different testing environments. By conducting finite element predictions of lattice hydrogen concentration, the influence of hydrostatic stress could be investigated to better compare the experimental results between different material strengths.

## Figures and Tables

**Table 1:** Chemical composition of AISI 4140 (DIN 1.7225, 42CrMo4) batch measured by optical emission spectroscopy.

			C	Si	Mn	P	S	Cr	Mo	Cu
Measured		[wt.%]	0.41	0.28	0.79	0.010	0.020	1.06	0.20	0.16
Standard	min		0.38	0.10	0.60	-	-	0.90	0.15	-
	max		0.45	0.40	0.90	0.025	0.035	1.20	0.30	0.40

**Table 2:** Hardness and quasi-static material properties from testing in air at room temperature as a reference.

Heat Treatment State	Hardness HV10	Yield Strength R_e_ [MPa]	Ultimate Strength R_m_ [MPa]
Medium strength (MS), 650 °C	320	877	969
High strength (HS), 400 °C	496	1455	1619

## Data Availability

The raw data supporting the conclusions of this article will be made available by the authors on request due to an ongoing study.
